# Three miRNAs cooperate with host genes involved in human cardiovascular disease

**DOI:** 10.1186/s40246-019-0232-4

**Published:** 2019-08-29

**Authors:** Yan Zhu, Jingjing Xie, Hong Sun

**Affiliations:** 1 Department of Cardiology, Gansu Provincial Hospital, Lanzhou, 730000 China; 2 Shanghai Children’s Hospital, Shanghai Jiao Tong University, Shanghai, 200062 China

## Abstract

**Background:**

Understanding the roles of miRNAs in cardiovascular disease remains a challenge. Genomic linkage indicates a functional relationship between intronic miRNAs and their host genes. However, few studies have shown functional association between intronic miRNAs and their host coding genes that are genetically associated with cardiovascular disease.

**Methods:**

In this study, we investigated functional relationship between three protein-coding genes genetically associated with cardiovascular disease, i.e., CDH13, SLC12A3, and CKAP5, and their intronic miRNAs using a data-driven approach.

**Results:**

We found that the three protein-coding genes functionally interact with targets of their intronic miRNAs, i.e., miR-3182, miR-6863, and miR-5582, in a tissue-specific pattern. The intronic miRNAs preferentially impact important genes for the three host genes in the network, indicating their roles in maintaining the integrity of the interactome where the host genes are involved. Targets of the intronic miRNAs display functional similarity to the host genes. We furthermore present sets of target genes for future investigation on the possible miRNA-target interactions that potentially contribute to cardiovascular diseases.

**Conclusions:**

Our work provides new insight into the regulatory network of the cardiovascular-associated pathways and opens the possibility for future experimental research.

**Electronic supplementary material:**

The online version of this article (10.1186/s40246-019-0232-4) contains supplementary material, which is available to authorized users.

## Introduction

The connection between miRNAs and disease was demonstrated to be obvious [1–4]. miRNAs have been implicated in a wide variety of cardiovascular disorder, including heart failure, cardiac hypertrophy, remodeling after myocardial infarction, arrhythmias, atherosclerosis, atrial fibrillation, and peripheral artery disease [5, 6]. Studies have shown that miRNAs play important roles in cardiac signaling and transcriptional pathways and that they act as “rheostats” and “switches” to modulate various aspects of cardiac development [7, 8]. Deregulated miRNA expression profiles were detected in patients with cardiac vascular disease [9, 10]. Functions in cardiac-associated pathways for some miRNAs have been verified, e.g., MiR-18a-5p inhibits endothelial-mesenchymal transition and cardiac fibrosis through the Notch2 pathway [7], and some miRNAs act as gatekeepers of cardiac cell functions by repressing deleterious targets [8]. Despite these advances in recent years, many questions remain regarding the mechanistic basis of miRNA activities, and numerous issues must be addressed toward their roles in cardiovascular disease.

Characterizing the influence of miRNAs in the context of targets is critical to better understand how miRNAs influence disease processes. However, the regulatory effects of miRNAs on targets are difficult to characterize because each miRNA has multiple mRNA targets and also because genes with tissue-specific function play key roles in the physiological processes of complex organisms [11]; therefore, correct identification of miRNA-target interactions remains a challenge. Elucidation of miRNA function requires technologies including bioinformatics prediction algorithms, reporter assays, in situ hybridizations, overexpression, and silencing technologies [12]. Using experimental methods to identify associations between miRNAs and diseases is demanding and costly.

It has been reported that genomic linkage between intronic miRNAs and their host genes indicates a functional relationship [13]. This finding extends the ways of elucidating intronic miRNA functionality beyond common strategies, such as gene expression analysis. Instances of functional relationships between intronic miRNA gene and its host gene are known. For example, the MYH7b gene hosts the miR-499 gene, the transcription product of which targets the 3′ untranslated region of the transcription factor SOX6; the SOX6 gene in turn acts as a repressor of MYH7b transcriptional activity [14]. The miR-208 gene is located in the intron of the alpha myosin heavy chain (MHC) gene; MHC reacts to stress and hyperthyroidism by coexpressing with miR-208 [15], and miR-208 in turn downregulates the expression of beta MHC [16].

However, few studies have shown the functional interaction between intronic miRNAs and the host coding genes that genetically contribute to cardiovascular disease (CVD). As disordered interplays between genes in tissue-specific processes were frequently found in human diseases [17, 18], in this work, we investigated the functional relationship between three genes, which are genetically associated with CVD, and their intronic miRNAs with a data-driven approach based on a large-scale tissue-specific gene interaction data [11] and gene annotation data.

## Results

We focus on the human protein-coding genes genetically associated with CVD annotated by Eupedia (https://www.eupedia.com/genetics/heart_disease_snp.shtml) and by scientific publications [19–22] to investigate the links between miRNAs and the molecular mechanism of CVD. A brief overview of the study is shown in Additional file 1: Figure S1. Around a hundred genes were found containing inherited DNA sequence variants which play a role in conferring risk for the CVD disease (CVD genes for short). Of all the human miRNAs annotated in miRBase [23], around 64% are located in introns in sense or antisense orientation to the host coding genes. Function of intronic miRNAs has the potential to be connected to their host genes by regulating target gene expression [13]. To investigate potential effects on CVD from miRNAs, we focused on the CVD genes that host miRNA gene. Among the set of protein-coding genes genetically related to CVD, we found three genes colocalize with miRNA, that is, CDH13 hosts miR-3182 (Additional file 1: Figure S2), SLC12A3 hosts miR-6863 (Additional file 1: Figure S3), and CKAP5 hosts miR-5582 (Additional file 1: Figure S4).

Studies have shown that mutations in the three protein-coding genes are associated with CVD. The CDH13 rs4783244 polymorphism confers stronger cardio-protection [21], and the CDH13 rs11646213 polymorphism is associated with risk of developing hypertension [22]. The SLC12A3 mutations produce clinically significant blood pressure reduction and protect from development of hypertension [20, 24]. The CKAP5 rs10734548 polymorphism shows significant association with ischemic stroke phenotype [19].

All the three human miRNA genes analyzed here are poorly conserved (Additional file 1: Figures S2–S4). Only the human miR-3182 gene has an ortholog, its ortholog appears only in the gorilla genome, and the host gene CDH13 and intronic miR-3182 pair is conserved between the two genomes (Additional file 1: Figure S2B). The three miRNA genes are evolutionarily young. Lineage-specific miRNAs may contribute to developmental novelties during evolution [25]; hence, it is worthwhile to study these non-conserved or human-specific miRNA genes.

### The intronic miRNAs show functional relationship with the CVD genes

Function of intronic miRNAs has the potential to be connected to their host genes by regulating target gene expression [13]. For the predicted targets of miRNAs, transcriptional expression analysis and network context based filters are effective in reducing false-positive rates [26–28]. Based on these, the data generated by Greene et al. [11] was used to filter the computationally predicted targets as the dataset was inferred by integrating tissue-specific gene expression data and protein-protein interaction data. We, therefore, investigated the functional relationship between the three CVD genes and the putative targets of their intronic miRNAs by employing the network-wide association study (NetWAS) scores [11]. Briefly, the NetWAS score evaluates the functional relationship between each pair of genes according to a posterior probability of tissue-specific functional information. In this work, we assessed five tissue-specific networks, i.e., three tissues that are directly related to CVD (cardiac muscle, heart, and vascular endothelium) and two tissues that are less obviously concerned (hair follicle and skin).

**Fig. 1:**
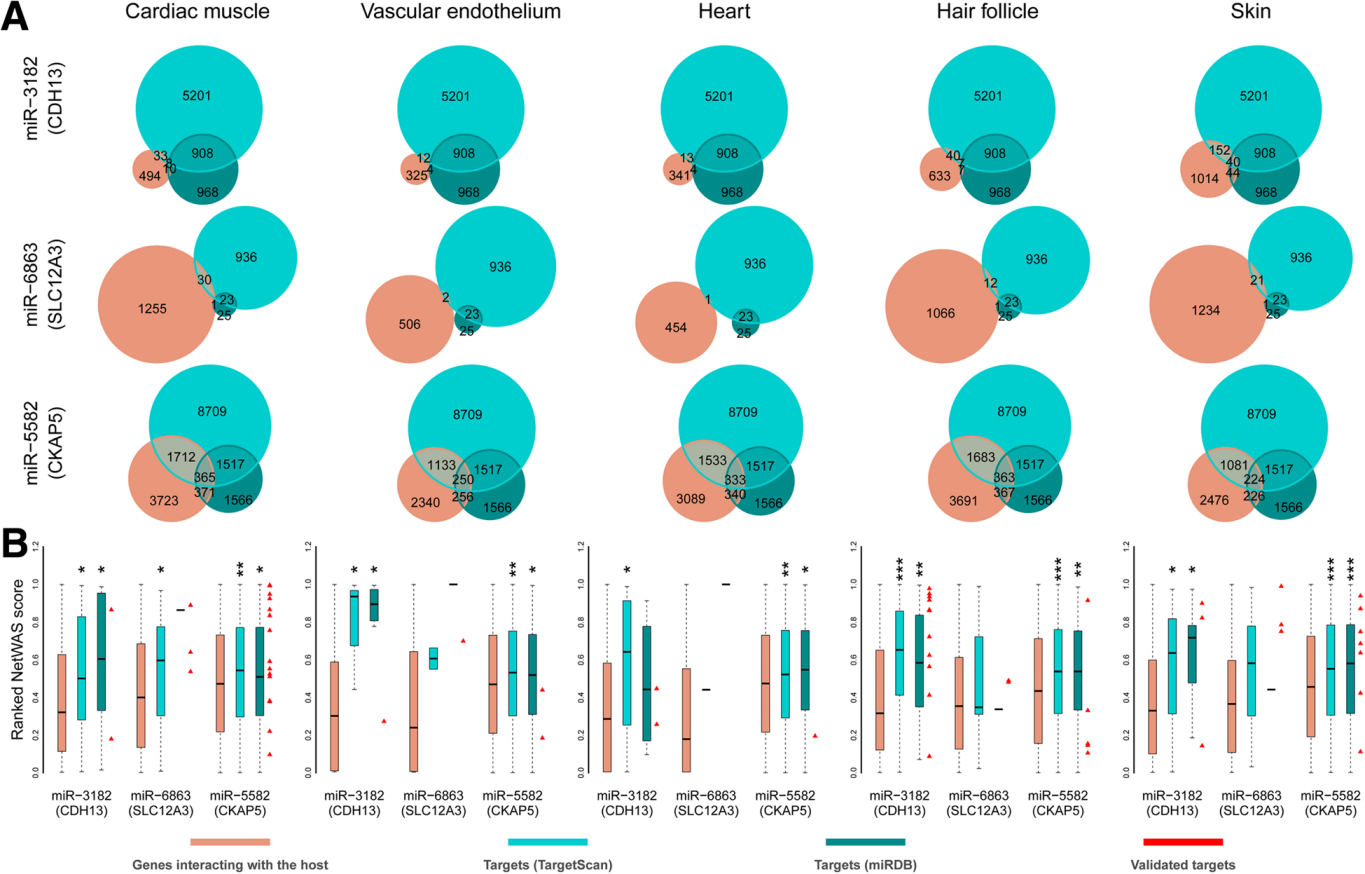
Targets of the intronic miRNAs show functional correlation to the CVD genes. **a** Venn diagram showing the number of overlapping between genes in association with the host gene and the targets of the intronic miRNA predicted by TargetScan and miRDB. **b** Box plots represent the ranked NetWAS scores underlying the degree of the association between the CVD gene and all the genes interacting with the host gene, and the degree of the association between the CVD gene and the predicted targets of its intronic miRNAs. Validated targets are represented by red triangle. Wilcoxon’s signed rank test is used to test the significance of differences between targets and all genes interacting with the host gene. *P < 0.05, **P < 10^−5^, ***P < 10^−10^

The predicted targets were screened through the five tissue-specific NetWAS networks, and only those which interact with the host gene were left for further analysis. The filtered targets reduce sharply (Fig. 1a). None of the host genes are predicted targets of their own intronic miRNAs*.* We found that the number of targets interacting with the host gene varies among different tissue networks (Fig. 1a).

Targets of the intronic miRNAs show clear functional relationships to the CVD genes. As seen in Fig. 1b, for the host gene CDH13 and CKAP5, the NetWAS scores of the target genes are significantly higher compared to the scores of all the associated genes in all the five tissue networks. As for the CVD gene, SLC12A3, the differences are not significant; one reason is that few targets are functionally correlated to SLC12A3 so that no statistical tests were performed because too few data were available to make a reliable comparison.

### The CVD genes interact with targets of their intronic miRNAs in a tissue-specific pattern

We examined the statistical significance of the overlap between predicted targets and genes with a functional relationship to the host gene. Despite the large number of predicted targets, the overlap is significantly weaker than random expectation (*P* < 10^−22^, hypergeometric test, Fig. 1a), but not entirely absent in all the cases.

**Fig. 2:**
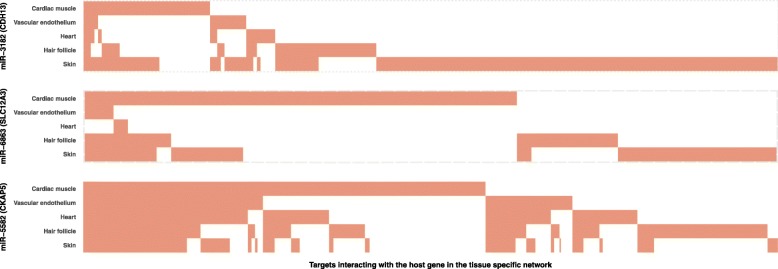
Sets of targets interacting with the host gene vary in different tissue networks

In addition, we found that in most cases, different sets of targets are associated with the host CVD genes in different tissue networks. Most targets in association with the host CVD gene were found in only one tissue network (Fig. 2), indicating that the corresponding intronic miRNA may modulate the function of its host gene in a tissue-specific pattern. The most visible differences are seen in the cardiac muscle network and in the skin network (Fig. 2).

### The intronic miRNAs impact core genes for the CVD gene in the network

**Fig. 3:**
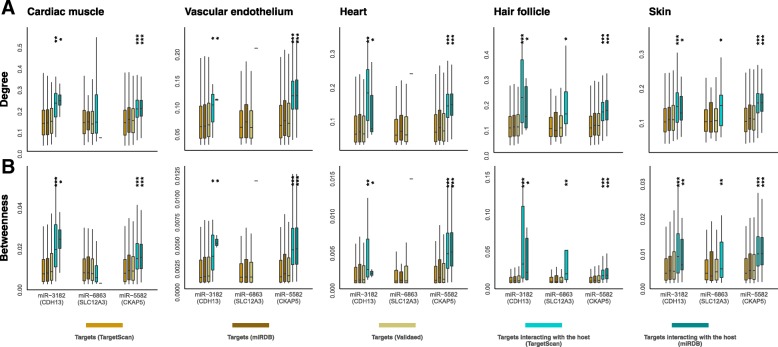
Network properties of the targets of the intronic miRNAs. Degree (**a**) and betweenness (**a**) distribution of targets in the tissue-specific networks. Wilcoxon’s signed rank test is used to test the significance of differences between all predicted targets and those interacting with the host gene. *P < 0.05, **P < 10^−5^, ***P < 10^−10^

We next explored the impact of the intronic miRNAs on the interactions with the host gene. We first compared the network degree of the targets interacting with the host gene to the network degree of all the predicted targets. We found that targets interacting with the host gene on average have a significantly higher network degree over all the targets predicted (Fig. 3a). Likewise, when we analyzed betweenness, another measure of network centrality based on shortest paths, we observed similar tendencies (Fig. 3b). This suggests that these intronic miRNAs preferentially impact core genes for the three CVD genes in the network and thus play a central role in maintaining the integrity of the tissue-specific interactome where the CVD genes are involved.

### Targets of the intronic miRNAs display functional similarity to the CVD genes

It was suggested that effects achieved by miRNA-mediated knockdown may perturb the pathway or biological process activated by the host gene, or adjust the protein expression levels of intronic miRNA targets toward intended optimal concentrations [13]. From this perspective, for each of the CVD genes, we further analyzed the functional relationship between the host genes and putative targets of their intronic miRNAs from a tissue-specific viewpoint and with a knowledge-based approach. We screened the predicted targets with high ranked NetWAS scores (≥ 0.5) to the host, and then, the top 20 targets were used for the functional enrichment analysis (Additional file 1: Tables S1–S3, experimentally validated targets [29–31] are highlighted with a star). Gene set enrichment analysis (GSEA) [32] identified several enriched gene sets (Table 1).

**Table 1:** Significantly enriched gene sets

Tissue	Gene set name: description	FDR *q* value
miR-3182, CDH13		
Cardiac muscle	Go_intracellular_vesicle: any vesicle that is part of the intracellular region.	0.0172
	Kim_bipolar_disorder_oligodendrocyte_density_corr_up: genes whose expression significantly and positively correlated with oligodendrocyte density in layer VI of BA9 brain region in patients with bipolar disorder.	0.0172
	Go_trans_golgi_network_membrane: the lipid bilayer surrounding any of the compartments that make up the trans-Golgi network.	0.0172
	Module_12: spinal cord (neurodevelopment) genes.	0.0274
	Go_regulation_of_cardiac_muscle_cell_contraction: any process that modulates the frequency, rate, or extent of cardiac muscle cell contraction.	0.0274
	Go_regulation_of_cardiac_muscle_cell_contraction: any process that modulates the frequency, rate, or extent of cardiac muscle cell contraction.	0.0274
Vascular endothelium	Baker_hematopoiesis_stat3_targets: selected genes downregulated in response to the Ras inhibitor salirasib in a panel of cancer cell lines with constantly active HRAS.	0.027
	Go_angiogenesis: blood vessel formation when new vessels emerge from the proliferation of pre-existing blood vessels.	0.04
miR-6863, SLC12A3		
Cardiac muscle	GO_NEURON_PART: any constituent part of a neuron, the basic cellular unit of nervous tissue.	0.048
miR-5582, CKAP5		
Cardiac muscle	Simbulan_parp1_targets_dn: genes downregulated in embryonic fibroblast cells from PARP1 knockout mice.	9 × 10^−10^
	Pujana_chek2_pcc_network: genes constituting the CHEK2-pcc network of transcripts whose expression positively correlates with that of CHEK2.	2 × 10^−9^
Vascular endothelium	Blum_response_to_salirasib_dn: selected genes downregulated in response to the Ras inhibitor salirasib in a panel of cancer cell lines with constantly active HRAS.	5 × 10^−27^
	Markey_rb1_acute_lof_up: Genes upregulated in adult fibroblasts with inactivated RB1 by cre-lox: acute loss of function of RB1.	5 × 10^−25^
Heart	Blum_response_to_salirasib_dn: selected genes downregulated in response to the Ras inhibitor salirasib in a panel of cancer cell lines with constantly active HRAS.	5 × 10^−25^
	Berenjeno_transformed_by_RHOA_up: genes upregulated in fibroblast cells transformed by expression of constitutively active (q63l) form of RHOA off plasmid vector.	3 × 10^−20^

Gene CDH13 encodes a member of the cadherin superfamily. The encoded protein protects vascular endothelial cells from apoptosis due to oxidative stress [33]. Eighteen targets of its intronic miR-3182, which interact with CDH13 gene in the cardiac muscle network, rank high in NetWAS scores (Additional file 1: Table S1). Enriched gene sets are shown in Table 1. GSEA analysis shows that the function of these targets is enriched in the process that modulates the frequency, rate, or extent of cardiac muscle cell contraction.

In the vascular endothelium network, 11 targets of miR-3182, which show association with the host CDH13 gene, rank high in NetWAS score (Additional file 1: Table S1). As shown in Table 1, these targets are enriched in several gene sets including genes downregulated in response to the Ras inhibitor salirasib. Evidence has placed Ras signaling at the center of pathways for a diverse subset of cardiovascular diseases, including cardiac hypertrophy and failure, angiogenesis, and endothelial dysfunction [34]. The target gene CCND2 ranks high among the genes connected to the host CDH13 gene in the vascular endothelium network (Additional file 1: Table S1), and it was reported that mouse mutant phenotypes for CCND2 include cardiovascular system phenotype [35].

Gene SLC12A3 encodes a renal thiazide-sensitive sodium-chloride cotransporter, and the protein is the target for thiazide diuretics which is used for treating high blood pressure [33]. Eighteen targets of its intronic miR-6863, which show an association with SLC12A3, rank high in NetWAS score in the cardiac muscle network, and they were used for the functional analysis (Additional file 1: Table S2). These targets are enriched in the gene set functioning in neuron (Table 1). It is worth mentioning that target gene RBM28 ranks high among the genes connected to the host SLC12A3 gene (Additional file 1: Table S2), and it is an important gene associated with post-cardiac arrest syndrome [36].

Gene CKAP5 encodes a cytoskeleton-associated protein which belongs to the TOG/XMAP215 family [33]. Top 20 targets of its intronic miR-5582, which rank high in cardiac muscle networks, in vascular endothelium network, and in heart network, were used for GSEA analysis respectively (Additional file 1: Table S3). The enriched gene sets, shown in Table 1, include differentially regulated genes in fibroblast-associated studies and genes in response to the Ras signaling. The target gene AQP1 ranks high among the targets connected to CKAP5 in the cardiac muscle network (Additional file 1: Table S3), and it was reported that mouse mutant phenotypes for AQP1 include cardiovascular system phenotype [32].

## Discussion

It is known that majority of intronic miRNAs are generally coexpressed with their host genes [37] and mediate synergistic and antagonistic regulatory effects between their host genes and target genes [13]. However, the involvement of intronic miRNAs in human cardiovascular disease is still largely unknown. In this study, we demonstrated a functional cooperation between the intronic miRNA loci and the three host genes which are genetically associated with cardiovascular disease. We found that the three CVD genes, i.e., CDH13, SLC12A3, and CKAP5, interact with targets of their intronic miRNAs, i.e., miR-3182, miR-6863, and miR-5582, in a tissue-specific pattern, and that their target genes display functional similarity to the host CVD genes.

Functions have been described for the three studied miRNAs. Differential expression in disease states has been identified for miR-3182 [38, 39]. It has been reported that overexpression of miR-5582-3P affects TGFβ signaling through targeting [40] and that mirR-6863 is deregulated in FTO knockdown cells [41]. Assessment of the regulatory effects of miRNAs on targets relies on correct identification of miRNA-target interactions. In this study, we filtered the predicted targets to reduce false-positive rates of computational predictions by means of the NetWAS score data generated by Greene et al. [11]. Our data showed evidences that the three studied intronic miRNAs interact with different sets of targets in a tissue-specific way and that their targets are enriched in gene sets functionally connected to the corresponding CVD gene.

Studies have identified cooperative activities of host gene and intronic miRNA pairs in other diseases. For examples, increased activity of IGF2 has been associated with many types of cancer, and upregulated expression of its intronic miR-483-3p protects cells from apoptosis [42]. Host gene SREBP and its intronic miR-33 contribute cooperatively in metabolic diseases [43]. Studies have revealed that miRNA targets are often involved in similar biological functions or are close to each other in interaction networks [44, 45]. Based on these studies and our findings, we can suggest that the three intronic miRNAs participate in the regulation of the cellular functions of their host genes and thus play a role in the biological process of human cardiovascular disease.

miRNA target prediction methods are error prone, and there also exist problems in the experimentally validated targets derived from the validation databases, that is, they do not account for the tissue-specific characteristic of miRNA-target interaction. At these points, we can speculate about the true nature of miRNA-target interaction events, and meanwhile, further experiments are needed to test the hypotheses presented here. Combining strategies is required to obtain a comprehensive view of miRNA targeting as each methodology has its strengths and weaknesses [46]. To dissect those functional relationships in particular tissues that are directly related to CVD, it is also essential to perform systematic analysis of tissue-specific coexpression between the three intronic miRNA genes, their host genes, and their target genes.

Our results provide new insight into the regulatory network of the cardiovascular-associated pathways and open the possibility for future experimental research. Although future validation experiments are needed, we believe our analysis assists in predicting specific sets of targets of these three studied miRNAs to investigate cardiovascular health in a tissue-specific manner.

## Conclusion

The data suggests that the three intronic miRNAs participate in the regulation of the cellular functions of the corresponding CVD genes in a tissue-specific way. Our findings provide new insight into the regulatory network of the cardiovascular-associated pathways and open the possibility for future experimental research.

## Materials and methods

### Data

The human coding genes genetically associated with cardiovascular disease were extracted from annotations by Eupedia (https://www.eupedia.com/genetics/heart_disease_snp.shtml), from review article by Kathiresan and Srivastava [20] and scientific publications [19, 21, 22].

Pre-assembled datasets for miRNA to mRNA interactions in the human genome predicted by TargetScan [47] and by miRDB [48] were downloaded from miRBase (release 21) [49]. The miR-5582 locus appears able to express two mature miRNA sequences, and the predicted targets of the two mature sequences are integrated together into the analysis. Validated miRNA-target interactions were downloaded from three databases*, i.e.,* miTarBase [29], miRNet [30], and miRWalk [31]. We integrated the data from the three different validation databases together into analysis because of the small number of validated interactions.

To reprioritize functional associations from a genome-wide association study, we downloaded the NetWAS scores via http://giant.princeton.edu/download/ that are introduced by Greene et al. [11] and we assessed five tissue-specific networks including cardiac muscle, heart, vascular endothelium, hair follicle, and skin.

### Conservation analysis

The UCSC Genome Browser [50] was used to inspect evolutionary conservation across 30 mammals as measured by PhastCons and PhyloP score [51]. We searched the miRBase [49] and Ensembl database [52] to find orthologs of the three miRNA genes.

### Evaluation of the essentiality of the target genes using measures of network centrality

We calculated the degree scores of network nodes and betweenness centrality to the extent to which a gene lies on paths between other genes [53]. For comparison, we standardized the degree centrality by dividing by the maximum possible degree expressed as a percentage and the betweenness values as well. Wilcoxon’s signed rank test is used to test the significance of differences between all targets and those interacting with the host gene.

### Functional enrichment of miRNA targets

As miRNA seed sequences are small and have only limited complementarity to their target sites, even small differences in algorithms would produce a great diversity in target predictions. The miRNA target prediction techniques not only have high false-positive rate but also have high false-negative rates [54]. To reduce false-negative rate, we integrated predicted targets from TargetScan [47] and miRDB [48] together. It has been reported that gene expression analysis and network context-based filters are effective in reducing false-positive rates [26–28]. To reduce false-positive rate, the data generated by Greene et al. [11] were used to filter the computationally predicted targets because the set of data is inferred by integrating tissue-specific gene expression data, protein-protein interactions, and tissue-specific information.

To reduce the risk for comparing NetWAS scores made up in different tissue networks, we replaced each observation by its fractional rank, the rank of NetWAS score divided by the total number of genes associated with the host gene. We further screened targets with high ranked NetWAS scores (≥ 0.5), and then, the top 20 targets were used for the functional enrichment analysis. We used the gene set enrichment analyses (GSEA) to test for enrichment in 17,810 gene sets from MSigDB (v6.2) [32, 55, 56]. *P* values were adjusted by FDR.

Data extraction and organization were performed by in-house perl scripts, and the statistical tests were performed using the R package.

## Additional file

Additional file 1: **Figure S1.** Schematic representation of the study design. **Figure S2.** Genomic location and sequence conservation of the human miR-3182 gene. A, Evolutionary conservation across 30 mammals is measured by PhastCons and PhyloP score. B, Pairwise alignment between the human miR-3182 gene and its gorila ortholog. **Figure S3.** Genomic location and sequence conservation of the human miR-6863 gene. Evolutionary conservation across 30 mammals is measured by PhastCons and PhyloP score. **Figure S4.** Genomic location and sequence conservation of the human miR-5582 gene. Evolutionary conservation across 30 mammals is measured by PhastCons and PhyloP score. **Table S1.** Targets of miR-3182 used for GSEA analysis. The NetWAS scores, which describe the probability of interaction between the host gene CDH13 and the predicted targets of miR-3182, are replaced by their fractional ranks. Validated targets are highlighted with a star. **Table S2.** Targets of miR-6863 used for GSEA analysis. The NetWAS scores, which describe the probability of interaction between the host gene SLC12A3 and the predicted targets of miR-5582, are replaced by their fractional ranks. Validated targets are highlighted with a star. **Table S3.** Targets of miR-5582 used for GSEA analysis. The NetWAS scores, which describe the probability of interaction between the host gene CKAP5 and the predicted targets gene of miR-5582, are replaced by their fractional ranks. Validated targets are highlighted with a star. (DOC 6113 kb)

## Data Availability

Data sharing is not applicable to this article as no datasets were generated or analyzed during the current study.

## Abbreviations

CVD: Cardiovascular disease
NetWAS: Network-wide association study
