# Supplementary material for: Three miRNAs cooperate with host genes involved in human cardiovascular disease
Source: Hum Genomics. 2019 Aug 29;13:40. doi: 10.1186/s40246-019-0232-4 (PMC6714460; doi:10.1186/s40246-019-0232-4)
Supplement: Supplementary file 1 — Figure S1. Schematic representation of the study design. Figure S2. Genomic location and sequence conservation of the human miR-3182 gene. A, Evolutionary conservation across 30 mammals is measured by PhastCons and PhyloP score. B, Pairwise alignment between the human miR-3182 gene and its gorila ortholog. Figure S3. Genomic location and sequence conservation of the human miR-6863 gene. Evolutionary conservation across 30 mammals is measured by PhastCons and PhyloP score. Figure S4. Genomic location and sequence conservation of the human miR-5582 gene. Evolutionary conservation across 30 mammals is measured by PhastCons and PhyloP score. Table S1. Targets of miR-3182 used for GSEA analysis. The NetWAS scores, which describe the probability of interaction between the host gene CDH13 and the predicted targets of miR-3182, are replaced by their fractional ranks. Validated targets are highlighted with a star. Table S2. Targets of miR-6863 used for GSEA analysis. The NetWAS scores, which describe the probability of interaction between the host gene SLC12A3 and the predicted targets of miR-5582, are replaced by their fractional ranks. Validated targets are highlighted with a star. Table S3. Targets of miR-5582 used for GSEA analysis. The NetWAS scores, which describe the probability of interaction between the host gene CKAP5 and the predicted targets gene of miR-5582, are replaced by their fractional ranks. Validated targets are highlighted with a star. (DOC 6113 kb) [file 40246_2019_232_MOESM1_ESM.doc]

**Supplemental materials for**

**Three miRNAs cooperate with host genes involved in human cardiovascular disease**

Yan Zhu ^1^, Jingjing Xie ^2^* and Hong Sun ^2^*

^1^ Gansu Provincial People's Hospital, Lanzhou University, Gansun 730000, China

^2^ Shanghai Children's Hospital, Shanghai Jiao Tong University, Shanghai 200062, China

*****Hong Sun, E-mail: sunhong@scbit.org

*****Jingjing Xie, xiejingjingcy@163.com

This file includes supplemental figures S1 to S4 and supplemental tables S1 to S3.

**Supplementary figures:**

Human coding genes genetically associated with CVD

CDH13, miR-3182

SLC12A3, miR-6863

CKAP5, miR-5582

**Host intronic miRNA gene(s)?**

Targets predicted by TargetScan and miRDB

Targets associated with the host gene

**Targets screening using GIANT data**

Functional importance of the targets

Functional association between the host genes and the targets

Targets have higher NetWAS Score

Functional similarity:

GSEA analysis & Cases

Network analysis: Higher degree and betweenness

**Figure S1:** Schematic representation of the study design.

**
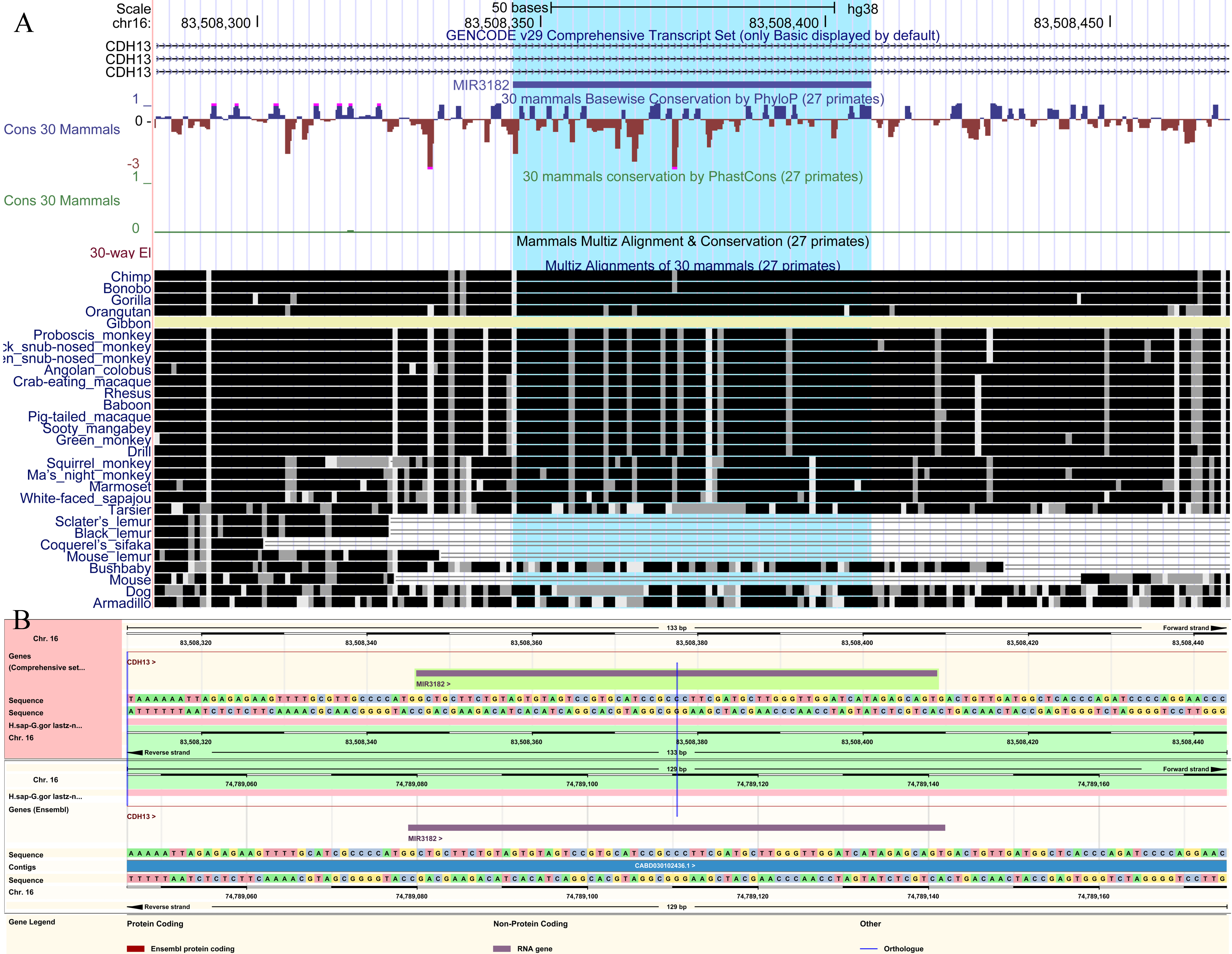
**

**Figure S2:** Genomic location and sequence conservation of the human miR-3182 gene. **A**, Evolutionary conservation across 30 mammals is measured by PhastCons and PhyloP score. **B**, Pairwise alignment between the human miR-3182 gene and its gorila ortholog.

**
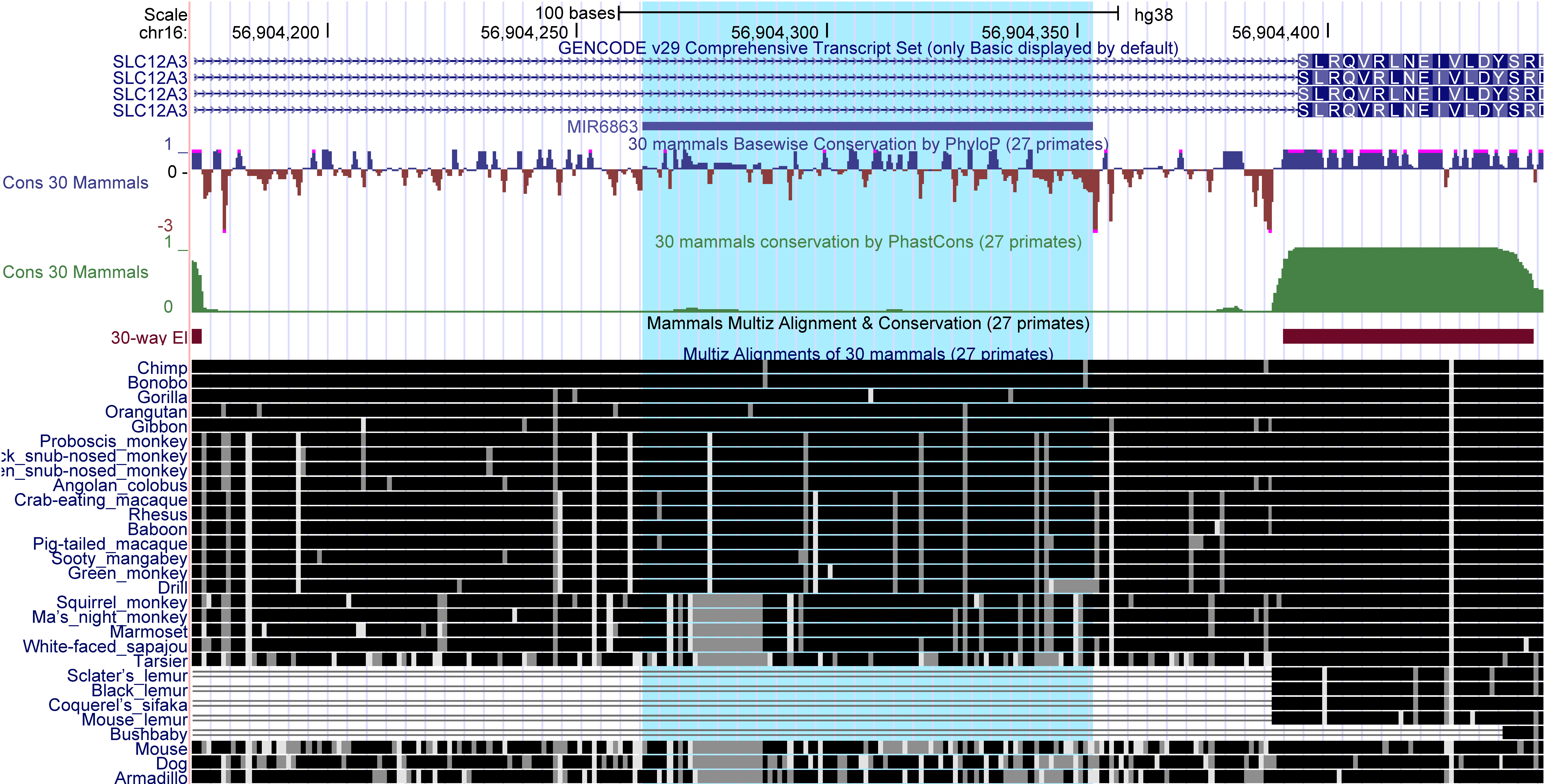
**

**Figure S3:** Genomic location and sequence conservation of the human miR-6863 gene. Evolutionary conservation across 30 mammals is measured by PhastCons and PhyloP score.

**
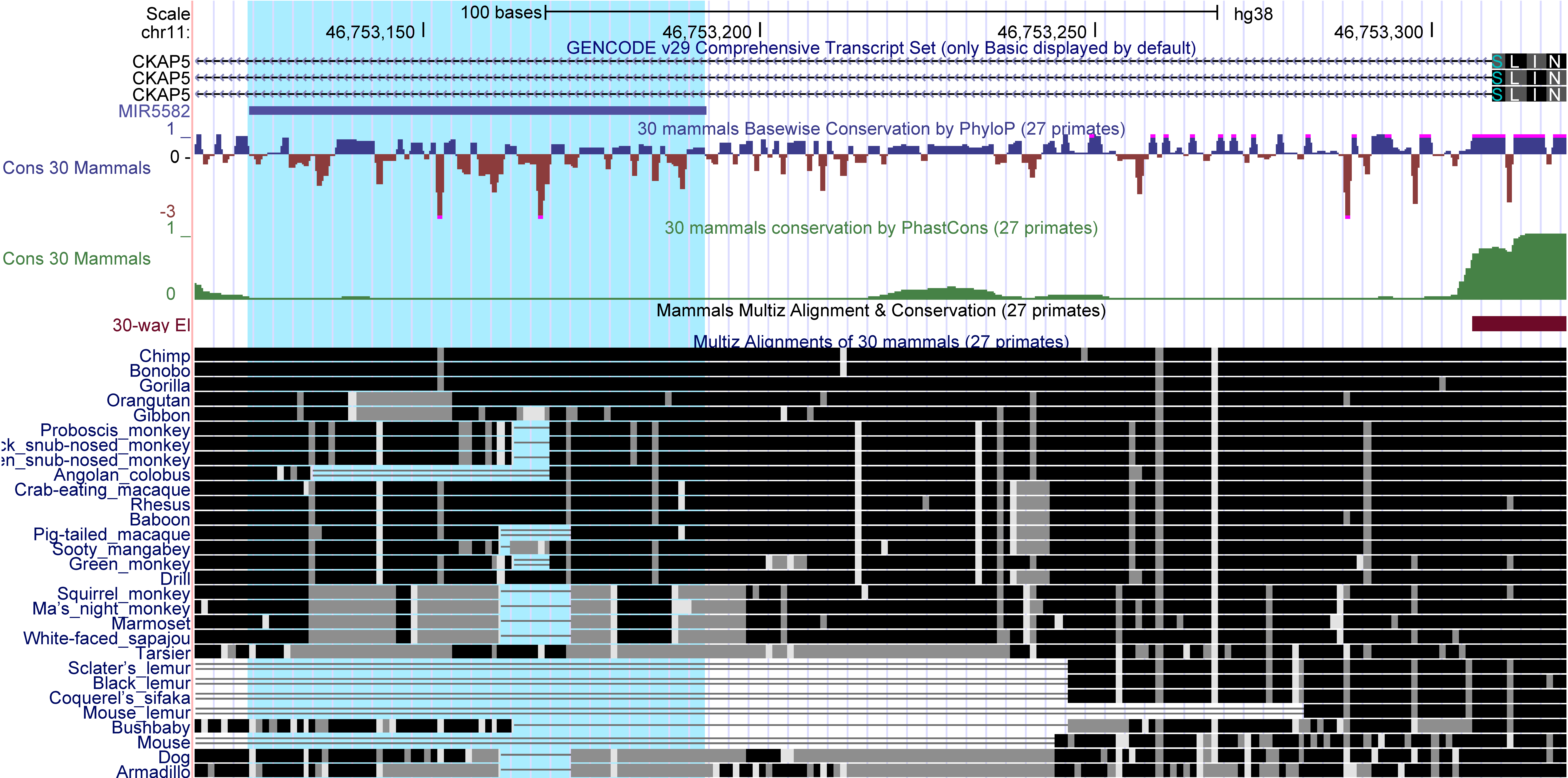
**

**Figure S4:** Genomic location and sequence conservation of the human miR-5582 gene. Evolutionary conservation across 30 mammals is measured by PhastCons and PhyloP score.

**Supplementary tables:**

**Table S1: Targets of miR-3182 used for GSEA analysis. The NetWAS scores, which describe the probability of interaction between the host gene CDH13 and the predicted targets of miR-3182, are replaced by their fractional ranks. Validated targets are highlighted with a star.**

| Cardiac muscle | | Vascular endothelium | |
| --- | --- | --- | --- |
| Target | Ranked NetWAS score | Target | Ranked NetWAS score |
| SUB1 | 0.993808 | CCND2 | 0.994595 |
| CCND2 | 0.987616 | DAB2IP | 0.983784 |
| ARHGEF37 | 0.959752 | PAPPA | 0.972973 |
| MXRA7 | 0.956656 | C11orf96 | 0.962162 |
| TGOLN2 | 0.95356 | SGTB | 0.951351 |
| DEFB107A | 0.922601 | DEFB107A | 0.945946 |
| SRI | 0.891641 | PTPRD | 0.924324 |
| MAGEB10 | 0.829721 | NOTCH3 | 0.918919 |
| VDAC1 | 0.811146 | MMP2 | 0.908108 |
| BCAP29* | 0.79257 | MAP4 | 0.837838 |
| CENPB | 0.743034 |  |  |
| VEGFC | 0.708978 |  |  |
| DAZAP2 | 0.622291 |  |  |
| AP1S2 | 0.588235 |  |  |
| ATP2A2 | 0.582043 |  |  |

**Table S2: Targets of miR-6863 used for GSEA analysis. The NetWAS scores, which describe the probability of interaction between the host gene SLC12A3 and the predicted targets of miR-5582, are replaced by their fractional ranks. Validated targets are highlighted with a star.**

| Cardiac muscle | |
| --- | --- |
| Target | Ranked NetWAS score |
| RBM28 | 0.967611 |
| LGI4 | 0.948381 |
| SSH3 | 0.902834 |
| GRIK3 | 0.897773 |
| LIPG | 0.866397 |
| CYP24A1 | 0.86336 |
| PARD6G* | 0.859312 |
| CAPRIN2 | 0.776316 |
| GPRIN1 | 0.774291 |
| DAB1 | 0.772267 |
| SPRED3 | 0.765182 |
| PARD6B | 0.73583 |
| TFAP2A | 0.709514 |
| MADCAM1 | 0.678138 |
| L3MBTL1 | 0.602227 |
| ZNF699 | 0.592105 |
| OPTC | 0.576923 |
| C10orf105 | 0.573887 |

**Table S3: Targets of miR-5582 used for GSEA analysis. The NetWAS scores, which describe the probability of interaction between the host gene CKAP5 and the predicted targets gene of miR-5582, are replaced by their fractional ranks. Validated targets are highlighted with a star.**

| Cardiac muscle | | Vascular endothelium | | Heart | |
| --- | --- | --- | --- | --- | --- |
| Target | Ranked NetWAS score | Gene | Ranked NetWAS score | Target | Ranked NetWAS score |
| AQP1 | 1 | CSE1L | 1 | AURKA | 1 |
| CEBPG | 0.99971 | CCNB1 | 0.999535 | RRM1 | 0.999645 |
| STMN1 | 0.998551 | RRM1 | 0.99814 | CSE1L | 0.998934 |
| BAG2 | 0.997101 | AURKA | 0.997675 | RFC5 | 0.998579 |
| CCNA2 | 0.996812 | CENPA | 0.996746 | CKS2 | 0.998224 |
| MRPL42 | 0.995942 | KIF11 | 0.996281 | DNMT1 | 0.997869 |
| PSMA4 | 0.994783 | CCNA2 | 0.995816 | RAD21 | 0.997513 |
| APPBP2 | 0.994203 | LRPPRC | 0.995351 | CCNA2 | 0.997158 |
| SERPINH1* | 0.993913 | CBFB* | 0.994421 | NUSAP1 | 0.996448 |
| KDM5B | 0.993333 | TOP2A | 0.993956 | CENPF | 0.996092 |
| NOLC1* | 0.993043 | DNMT1 | 0.993491 | KIF11 | 0.995382 |
| AKAP1* | 0.992464 | MAD2L1 | 0.992562 | PRKDC | 0.995027 |
| KIF11 | 0.991304 | CDKN3 | 0.992097 | TFDP1 | 0.994316 |
| HMGB2 | 0.990145 | CENPF | 0.990702 | SRSF10* | 0.993961 |
| PIAS2 | 0.989855 | STMN1 | 0.989772 | MCM4 | 0.99325 |
| ILF3 | 0.989275 | TFDP1 | 0.989307 | CCNB1 | 0.992895 |
| PHB | 0.988406 | RAD21 | 0.986518 | MSH2 | 0.992185 |
| PCNA | 0.987826 | NUSAP1 | 0.986053 | TACC1 | 0.991829 |
| PFKM | 0.987536 | CKS2 | 0.985588 | COPS3 | 0.991119 |
| TXNRD1 | 0.99913 | DLGAP5 | 0.985123 | TOP2A | 0.990764 |
